# PFAPA syndrome in the Czech Republic: a single-centre experience

**DOI:** 10.1186/1546-0096-10-S1-A85

**Published:** 2012-07-13

**Authors:** Petra Król, Marek Böhm, Dana Nemcová, Pavla Doležalová

**Affiliations:** 1 General University Hospital in Prague, Praha 2, Czech Republic

## Purpose

PFAPA syndrome (Periodic Fever, Aphthous stomatitis, Pharyngitis and Adenitis) is an idiopathic autoinflammatory disease with the first manifestation before 5 years of age. Fever attacks lasting for 3-6 days have individual periodicity within about 3-8 week intervals. They are accompanied by individual combination of other symptoms among which culture-negative pharyngitis/tonsillitis, oral aphthae and cervical adenitis are the most common. Children are healthy and thriving in between attacks. Clinical benign course with no long-term sequelae with normal growth and development is typical for PFAPA syndrome. Fever attacks usually do not regress with antibiotic therapy, but a single prednisone dose of 1mg/kg administered at the onset of fever has a dramatic effect. Tonsillectomy appears a promising curative method for more difficult PFAPA patients.

## Aim

To describe clinical and laboratory characteristics of a single-centre cohort of PFAPA patients.

## Methods

Retrospective chart review (patients diagnosed 2004-2007) and prospective collection (2008-2010) of clinical and laboratory data during febrile attack and in afebrile interval.

## Results

56 boys and 53 girls with PFAPA syndrome were diagnosed. Median age of the first manifestation was 20 months, median interval between attacks was 4 weeks and fever duration was 3,5 days. Patients were followed for median of 56,6 months. Fever was associated with pharyngitis/tonsillitis (87%), cervical adenitis (73%) and aphthous stomatitis (39%). Laboratory measures during the fever attack (median, range) were as follows: CRP (63,4; 12-237,4 mg/l), ESR (31; 9-60/h), WBC (14; range 3-20x10^9^/l). In all patients where follow-up values were available inflammatory parameters dropped back to normal within at least 2 weeks without fever. All patients had normal IgD levels and normal mevalonate in urine collected during the fever attack. A single prednisone dose was effective in about 90% of patients. Tonsillectomy lead to the complete remission in 12/13 children. Presence of self-limited recurrent fever with tonsillitis in early childhood of one of the parents was recorded in 45% of families.

## Conclusion

PFAPA syndrome appears to be a relatively common cause of recurrent fever in early childhood in the Czech Republic. Detailed analysis of clinical and laboratory data including long-term outcomes is ongoing.

## Disclosure

Petra Król: None; Marek Böhm: None; Dana Nemcová: None; Pavla Doležalová: None.

